# Dose–Response Study of Microcystin Congeners MCLA, MCLR, MCLY, MCRR, and MCYR Administered Orally to Mice

**DOI:** 10.3390/toxins13020086

**Published:** 2021-01-24

**Authors:** Neil Chernoff, Donna Hill, Johnsie Lang, Judith Schmid, Amy Farthing, Hwa Huang

**Affiliations:** 1 Center for Public Health and Environmental Assessment, Office of Research and Development, U.S. Environmental Protection Agency, Research Triangle Park, NC 27711, USA; Hill.Donna@epa.gov (D.H.); jeschmid@mebtel.net (J.S.); 2 Oak Ridge Institute for Science and Education, Oak Ridge, TN 37831, USA; johnsie.lang@arcadis.com (J.L.); alokichi@ncsu.edu (A.F.); hira719314@gmail.com (H.H.)

**Keywords:** harmful algal bloom, cyanobacteria, cyanotoxin, microcystin, hepatic toxicology, oral administration, mouse

## Abstract

Microcystins are common freshwater cyanobacterial toxins that affect liver function. The toxicities of five microcystin congeners (microcystin-LA (MCLA), MCLR, MCLY, MCRR, and MCYR) commonly observed in harmful algal blooms (HABs) were evaluated in BALB/c mice after a single oral administration of doses ranging from those that were no observed adverse effect levels (NOAELs) to lowest observed adverse effect levels (LOAELs). Animals were monitored for changes in behavior and appearance, and euthanized 24 h after dosing. Test endpoints included clinical changes, necropsy observations, and serum indicators of hepatic toxicity and general homeostasis. Doses were 0.5–7 mg/kg MCLA, 0.5–11 mg/kg MCLR, 1–7 mg/kg MCLY, 7–22 mg/kg MCRR, and 3–11 mg/kg MCYR. MCLA at 3 mg/kg elevated liver/body weight ratio and liver score, ALT, AST, and GLDH, indicating hepatic toxicity, reduced serum glucose and highly elevated total serum bilirubin. MCLR and MCLY induced similar effects with LOAELs of 5 mg/kg, although a greater extent and severity of effects were observed in MCLR animals. MCRR exposure at 22 mg/kg was associated with reduced serum glucose. MCYR induced scattered liver effects at 7 mg/kg and reduced serum glucose levels at 5 mg/kg. The results indicate significant differences in congener-induced toxicity after microcystin exposure.

## 1. Introduction

The phylum Cyanobacteria is comprised of photosynthetic Gram-negative bacteria that are ubiquitous in large numbers globally and extremely common in freshwaters. Cyanobacteria undergo periodic massive population size increases in freshwater systems, known as “blooms”. Many species are known to produce compounds that may be toxic to vertebrates and the rapid growth of these populations is referred to as harmful algal blooms (HABs) [1,2]. Blooms are thought to be caused by a combination of various factors including nitrogen and phosphorus concentrations [3,4,5], stability of the water column [6]; and increased water temperatures [7,8]. Multiple adverse vertebrate health effects are associated with HABs [9], including neurotoxicity from anatoxins and saxitoxins [10,11,12], and hepatic toxicity from cylindrospermopsin [13] and microcystins (MCs) [14,15]. The occurrence of MC-containing blooms in United States (U.S.) freshwaters has been well documented, and data indicate that MCs are the most commonly encountered cyanobacterial toxins globally [16].

MCs consist of two proteinogenic and five non-proteinogenic amino acids including 3-amino-9-methoxy-2,6,8-trimethyl-10-phenyl-4,6-decadienoic acid (ADDA) that has only been identified on these compounds and nodularin, a structurally related pentapeptide [17]. There are in excess of 240 MC structural variants [18], although the great majority of these are rarely encountered. The basis for many MC varieties lies in the identities and/or structures of the amino acids in the two proteinogenic amino acid-variable positions and the large number of structural variants in the other five amino acids, many of which are methylated and demethylated variants [19,20,21,22,23]. The basic nomenclature of the MCs is centered on the two variable amino acids—MCLR refers to leucine (L) and arginine (R), and MCLY refers to leucine and tryptophan (Y). The two most identified MCs in a survey of Midwestern U.S. algal blooms were MCLR and MCRR [24,25], but other MC congeners, including MCLA and MCYR, were predominant forms in some blooms. Dense blooms that are predominantly one type of MC may have high levels of other MCs. In one lake with a bloom primarily containing MCLR and MCRR, MCLA was present at concentrations of 54 µg/L, a level 140-fold greater than any other lake that contained it, including one where it was the primary toxin [24].

Most MC toxicology studies have involved MCRR or MCLR, especially the latter [26], because of its proven toxicity [27]. Studies using the intraperitoneal (i.p.) route of administration in mice have demonstrated that MCLR primarily affects the liver. Once in the blood, MCLR reaches the liver, where it is transported through the hepatocyte cell membranes by organic anion transporting polypeptides (OATPs), most notably OATP1B2 in the mouse and the orthologs OATP1B1 and OATP1B3 in humans [28,29,30]. In hepatocytes, MCLR induces cellular toxicity by inhibition of protein phosphatases 1 (PP1) and 2A (PP2A) resulting in hyperphosphorylation, associated disruption of the cytoskeleton, cellular breakdown, and cell death [31,32,33]. Metabolic formation of reactive oxygen species leads to oxidative stress, apoptosis, and DNA damage [34,35]. Acute exposure to high levels of MCs results in sinusoidal breakdown, intrahepatic bleeding, necrosis, and hemorrhagic shock [36,37].

The major route of exposure to HAB MCs in people is oral, either in water or food, although an incident involving MC-contaminated water supply in a dialysis center resulted in severe toxicity and death [38]. HABs produce a wide variety of MC congeners [18,39], but relatively few have been tested in vivo. Laboratory studies utilizing the appropriate oral route of administration are necessary for development of environmental exposure guideline values used to protect public health. To date, such studies have been limited to MCLR and MCRR. The additional MC congeners studied here, MCLA, MCLY, and MCYR, are found globally and have been reported from the U.S. [40], Europe [41], and Africa [42].

Studies on the toxicology of MCLR administered orally were reported by several investigators. Yoshida et al. [43] administered MCLR to mice and found a single-dose LD50 of 10.9 mg/kg. Fawell et al. [44] administered 0.04, 0.2, or 1.0 mg/kg by gavage to mice for 3 months and found significant elevations in markers of liver toxicity, serum alanine amino transferase (ALT) and aspartate amino transferase (AST) at doses ≥ 0.2 mg/kg. Lower serum albumin and total protein, inflammatory responses and hepatocyte degeneration were noted at 1.0 mg/kg. Heinze et al. [45] administered 0.05 or 0.1 mg/kg MCLR daily for 1 month to rats and found dose-related increases in liver weight, alkaline phosphatase (ALP), hepatocyte degeneration, and hemorrhages. Sedan et al. [46] investigated effects of a one-month exposure to 0.05 or 0.1 mg/kg MCLR administered to mice on alternate days and found increased cytoplasmic vacuolation and steatosis in the centrilobular zone and reduced superoxide dismutase activity in treated hepatocytes. Oral exposures of MCRR for 7 days at doses ≥ 0.046 mg/kg induced hepatic apoptosis although no effects on protein phosphatases were noted [47].

This paper summarizes data evaluating dose responses of single oral exposures to MCLA, MCLR, MCLY, MCRR, and MCYR in the BALB/c mouse using toxicity endpoints indicative of hepatic toxicity and altered homeostasis. It follows a previous paper [48] that compared the toxicities of ten MC congeners after exposures to the single-dose level of 7 mg/kg of one of the five congeners summarized here as well as MCLF, MCLW, [Asp3]MCRR, [Asp3,Dhb7]MCRR, and MCWR. The data in the completed comparative MC congener study were used to select a starting dose range for determining acute exposure NOAELs and LOAELs based on live animal and organ observations, body and organ weights, and changes in serum components indicative of liver function and general homeostasis. The 7 mg/kg data for the MCLA, MCLY, MCRR, and MCYR, as well as part of the MCLR dose–response data, were also presented in [48].

## 2. Results

All data are summarized in supplementary tables (in Supplementary Materials file): MCLA—Table S1, MCLR—Table S2, MCLY, Table S3, MCRR—Table S4, and MCYR—Table S5.

### 2.1. Moribundity, Liver Score, Liver/Body Weight Ratios and Serum Bilirubin Levels

Dose ranges varied across congeners. Significant moribundity incidence (hunching, non-responsiveness to interaction, lethargy, hypothermia, diarrhea, and/or weight loss greater than 10%) was recorded in the 7 mg/kg level of MCLA mice (*p ≤* 0.001 males; *p ≤* 0.01 females). Significant moribundity was seen in 7 mg/kg MCLR males (*p ≤* 0.05), and 9 and 11 mg/kg males and females (*p ≤* 0.01). Sporadic moribundity also occurred in the 7 mg/kg MCLY and the 7 and 11 mg/kg MCYR animals. Males were generally more affected than females, with higher incidences of moribundity in the 7 mg/kg MCLA and MCLR, and MCYR 11 mg/kg groups; and only males were affected in the 5 mg/kg MCLR and the 7 mg/kg MCLY and MCYR groups. Urine with a yellow tint was observed in both sexes of the MCLA animals in the 7 mg/kg groups.

**Figure 1:**
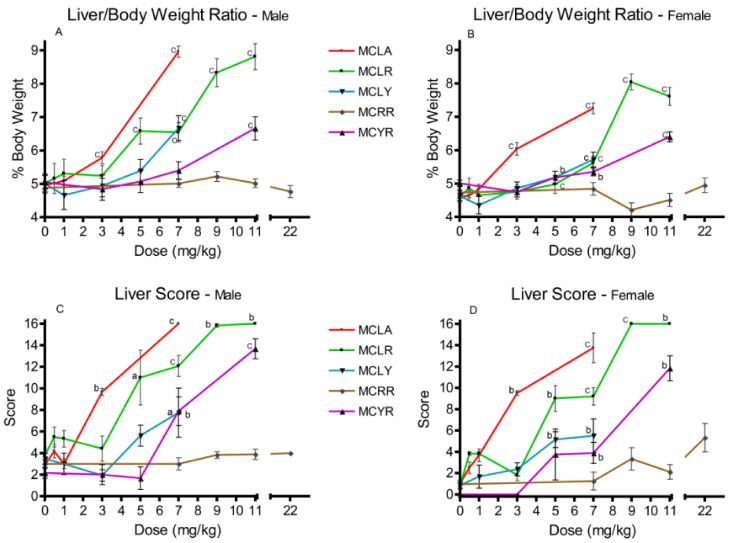
Effects in liver/body weight ratios and liver scores 24 h after exposure or at onset of moribundity following single administrations of different dose levels of MCLA, MCLR, MCYR, MCRR, or MCYR to BALB/c mice. Liver/body weight ratios ((**A**), males; (**B**), females) indicating increased relative liver weights and/or size of livers. Liver score ((**C**), males; (**D**), females), a general measure of the gross appearance of the organ at the time of necropsy; ^a^ *p* ≤ 0.05, ^b^ *p* ≤ 0.01. ^c^ *p* ≤ 0.001.

Elevated liver/body weight (L/BW) ratios were observed at 3 and 7 mg/kg with MCLA (*p ≤* 0.001); for MCLR males at 5 mg/kg and for both sexes in the 7–11 mg/kg groups. Comparable effects were observed for MCLY and MCYR females at 5 mg/kg (*p ≤* 0.05) and in both sexes at 7 mg/kg (*p ≤* 0.001) (Figure 1A,B). Significantly elevated liver scores were noted with the same range as the L/BW ratios. They occurred with effects noted at 3 and 7 mg/kg for MCLA; 5–11 mg/kg for MCLR; 5 and 7 mg/kg for MCLY; and at 7 and 11 mg/kg for MCYR (Figure 1C,D). MCRR exposures as high as 22 mg/kg did not affect either L/BW or liver scores in a significant dose-related manner.

### 2.2. Serum Markers of Liver Toxicity

**Figure 2:**
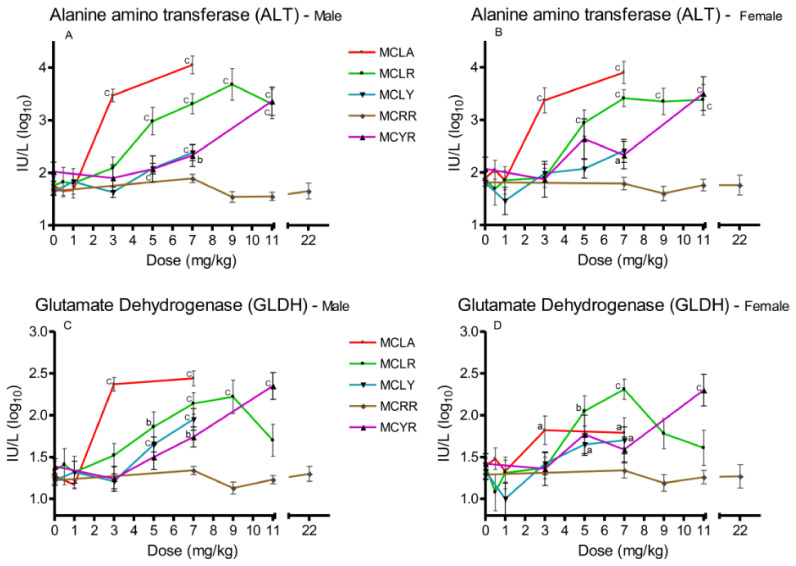
Changes in serum enzyme markers of hepatic toxicity 24 h after exposure, or at onset of moribundity, after single administration of different dose levels of MCLA, MCLR, MCYR, MCRR, or MCYR to BALB/c mice. Alanine aminotransferase (ALT) ((**A**), males; (**B**), females). Glutamate dehydrogenase (GLDH) ((**C**), males; (**D**), females); ^a^ *p* ≤ 0.05, ^b^ *p* ≤ 0.01. ^c^ *p* ≤ 0.001.

Three serum markers of hepatotoxicity were analyzed, ALT (Figure 2A,B), AST, and GLDH (Figure 2C,D). Significant (*p ≤* 0.05) elevations in all three were observed in the 3 and 7 mg/kg MCLA dose groups. MCLR animals exhibited significant increases (*p ≤* 0.001) in ALT and AST at 5–11 mg/kg; and significant (*p ≤* 0.01) GLDH levels at the 5–9 mg/kg dose levels in males and 5–7 mg/kg in females. MCLY animals had significantly (*p* ≤ 0.05) elevated levels of the three markers in males in the 5–7 mg/kg groups. Female animals had elevated (*p* ≤ 0.05) GLDH in the 5 mg/kg group and all serum markers were affected in both sexes receiving 7 mg/kg. MCYR animals had significantly elevated levels in GLDH in males (*p* ≤ 0.01) after exposure to 7 mg/kg and elevation of all three markers (*p* ≤ 0.001) at 11 mg/kg. MCRR animals’ serum markers were unaffected at all dose levels tested.

### 2.3. Serum Glucose, Total Protein, Blood Urea Nitrogen (BUN)/Creatinine Ratios, and Total Bilirubin

**Figure 3:**
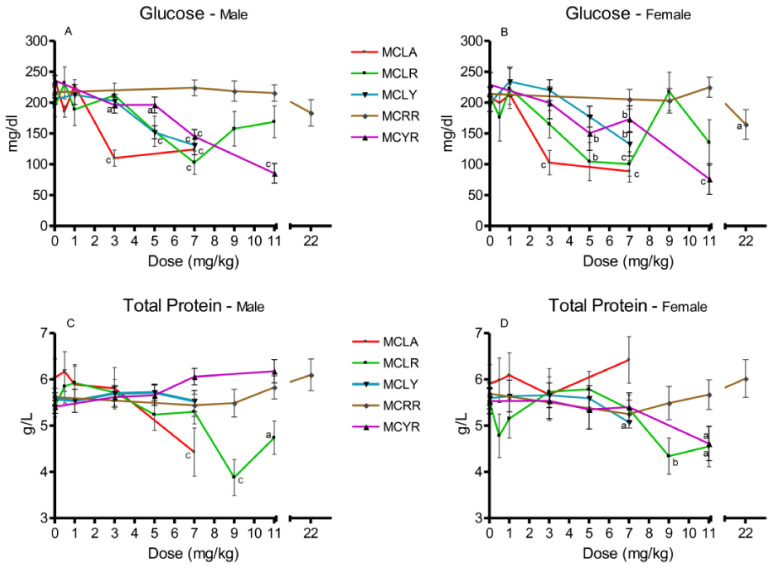
Changes in serum glucose and total protein 24 h after exposure, or at onset of moribundity, after single administration of different dose levels of MCLA, MCLR, MCYR, MCRR, and MCYR to BALB/c mice. Glucose ((**A**), males; (**B**), females). Total protein ((**C**), males; (**D**), females); ^a^ *p* ≤ 0.05, ^b^ *p* ≤ 0.01. ^c^ *p* ≤ 0.001.

Significant reductions in serum glucose were observed in MCLA groups exposed to 5 and 7 mg/kg (*p* ≤ 0.001) (Figure 3A,B). In the MCLR-treated animals, both sexes in the 7 mg/kg dose groups (*p* ≤ 0.001) and females exposed to 5 mg/kg (*p* ≤ 0.01) exhibited this effect. Reductions in serum glucose levels occurred in females at 7 mg/kg MCLY and males at 5 and 7 mg/kg. Significantly reduced serum glucose levels (*p* ≤ 0.05) were seen only in the 22 mg/kg MCRR females. Reduced glucose levels were a common finding in the MCYR animals, occurring in all 5–11 mg/kg dose groups and in the male 3 mg/kg group. Total serum protein (Figure 3C,D) was reduced in 7 mg/kg MCLA males (*p* ≤ 0.001). In MCLR animals, total serum protein was reduced in the 9 mg/kg (*p* ≤ 0.001) and 11 mg/kg (*p* ≤ 0.05) dose groups. The MCLY-dosed females had a decreased levels of total protein in the 7 mg/kg dose group (*p* ≤ 0.05). MCYR animals had significantly reduced levels of total protein in the 7 mg/kg males (*p* ≤ 0.01) and in both sexes receiving 11 mg/kg, (males (*p* ≤ 0.01) and females (*p* ≤ 0.05)).

**Figure 4:**
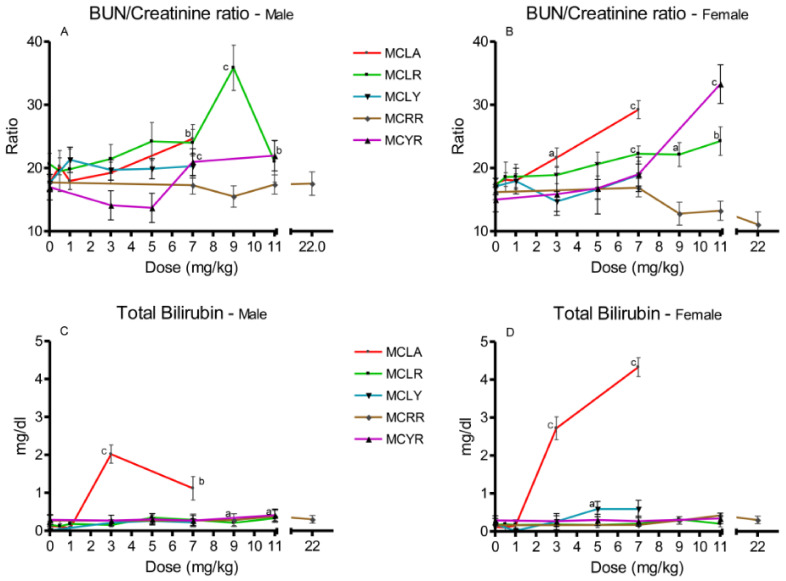
Changes in BUN/creatinine ratios and bilirubin levels 24 h after exposure, or at onset of moribundity, after single administration of different dose levels of MCLA, MCLR, MCYR, MCRR, and MCYR to BALB/c mice. BUN/creatinine ((**A**), males; (**B**), females). Total bilirubin ((**C**), males; (**D**), females); ^a^ *p* ≤ 0.05, ^b^ *p* ≤ 0.01. ^c^ *p* ≤ 0.001.

Increased BUN/creatinine ratios were noted for MCLA, MCLR and MCYR (Figure 4A,B). Elevated total serum bilirubin levels were noted in higher-dose groups of MCLR, MCLY, MCRR, and MCYR, with values ranging from 0.29 to 0.59 mg/dL (Figure 4C,D). Bilirubin levels in MCLA at doses of 3 and 7 mg/kg were significantly increased to concentrations ranging from 1.1 to 4.3 mg/dL.

### 2.4. Comparative Toxicity of Microcystin Congeners MCLA, MCLR, MCLY, MCRR, and MCYR and Determination of NOAELs

MCLA was the most toxic congener and the 3 mg/kg dose level induced significantly elevated L/BW, liver scores, and serum levels of ALT, AST, GLDH, and total serum bilirubin. The bilirubin levels were 10- to 40-fold greater than the comparable control values and yellow-tinted urine was only observed after exposure to this congener. Extensive moribundity after exposure to 7 mg/kg was observed (100% in males and 63% in females). Exposure to 1 mg/kg did not induce any toxic effects and may be considered the NOAEL for this congener.

MCLR had a similar array of effects as seen with MCLA, but 5 mg/kg was the LOAEL with significant elevations in multiple endpoints indicating hepatic toxicity at this dose level. There were significant 2- to 3-fold increases in total bilirubin at dose levels ≥5 mg/kg. Glucose was reduced in the 5 and 7 mg/kg dose levels, but not at higher dose levels. Total protein was reduced at dose levels where moribundity was present (9 and 11 mg/kg).

MCLY had scattered significant effects in hepatic endpoints as well as decreased glucose and increased bilirubin levels at the 5 (males) and 7 mg/kg levels (females). The LOAEL for MCLY, similar to MCLR, was 5 mg/kg.

MCRR was the least toxic congener of the ones reported here. The only significant effects were increased weight loss after exposures, and reduced serum glucose levels in the female group that received the highest dose level, 22 mg/kg.

MCYR exhibited a pattern of effects that appears to be different from MCLA, MCLR, and MCLY. Significant hepatic effects were observed only in animals exposed to the 7 and 11 mg/kg dose levels. Decreased glucose levels were noted at the 3 and 5 mg/kg dose levels but this could reflect the relatively high control levels of glucose in the concurrent controls for this group of test animals.

Changes in bilirubin levels in the MCLA group are consistent with jaundice and were not necessarily associated with moribundity. The smaller increases in bilirubin in other congeners were associated with moribundity in six of eight instances and may reflect general liver cell injury.

## 3. Discussion

The key findings, summarized in Table 1, are consistent with the conclusions about comparative MC congener acute toxicity reached in our previous 7 mg/kg single-dose study [48]. MCLA was the most toxic congener, with the 3 mg/kg LOAEL dose being hepatotoxic and also inducing significantly elevated BUN/creatinine ratios in females and decreased levels of serum glucose in both sexes. Very high levels of total bilirubin in the serum were seen at this dose level in the absence of clinical signs of moribundity, indicating the possibility that MCLA affects bilirubin production or excretion differently than other MCs. The yellow-tinted urine observed in the 7 mg/kg dose and the high levels of serum bilirubin are indicative of jaundice in the affected animals. Based on the data we have obtained, the 3 mg/kg dose is referred to as the LOAEL, but the severity of effects noted after exposure to this dose and the data gap between the 3 and 1 mg/kg NOAEL indicate the possibility that the actual LOAEL is significantly higher. As in the previous 7 mg/kg study, MCLR was also found to be highly toxic, with moribundity accompanied by significant hepatic toxicity occurring at 5 mg/kg, and a NOAEL of 3 mg/kg. MCLY also induced significant effects at 5 mg/kg, although fewer hepatic endpoints were significantly affected. The MCYR animals exhibited no significant liver effects at 5 mg/kg and only scattered significant effects at 7 mg/kg. Significantly decreased serum glucose levels were observed in females at 5 mg/kg and in both sexes at the 7 mg/kg. Statistically significant reductions in glucose levels were observed in males exposed to ≥3 mg/kg and this dose is therefore arguably the NOAEL for MCYR. The only significant toxicity observed for MCRR was reduced glucose levels in females exposed to 22 mg/kg. Based on these data, the NOAEL for MCRR would be 11 mg/kg in females and 22 mg/kg in males.

Both MCLR and MCLA have been associated with unexpected mortality events (UMEs) in the environment. MCLR is associated with UMEs in wildlife [49], livestock [50,51], and dogs [52,53]. MCLA has been associated with two instances of dog poisonings, with clinical findings that resemble those seen with MCLA in this study. Rankin et al. [54] reported an incident where an animal was exposed to MCLA in a HAB that contained 38,600 ppb of MCLA in the surface scum. The dog drank the pond water and subsequently ingested scum that was clinging to its fur. The animal exhibited dark yellow urine, icterus, and elevated liver enzymes indicative of jaundice and general hepatic toxicity. Bautista et al. [55] reported a case of poisoning in a 13.6 kg dog exposed to a calculated dose of ≈0.96 µg/day MCLA and ≈0.17 µg/day MCLR from a dietary supplement given continuously for 3.5 weeks prior to the appearance of symptoms indicative of hepatic dysfunction including elevated levels of ALT, AST, and total bilirubin in addition to other signs of general toxicity involving loss of appetite, lethargy, and polydipsia. The supplement was analyzed and 166 ng/g MCLR and 962 ng/g of MCLA were detected. MCLA may also have played a role in an UME involving sea otters off the coast of California, U.S.A [56]. Although there is no direct link between MCLA and the poisoning of these animals, the affected animals exhibited icterus, and the source of the MC accumulation in shellfish they subsist on is thought to be Pinto Lake, which was experiencing a large MCLA-producing HAB (2.9 million ppb) at that time.

The MC congeners investigated in this study were also identified in HABs reported in a study of 23 Midwestern lakes [24]. Water collected from the lakes contained different mixtures of MCs that included all congeners studied in this paper. MCLR and MCRR were the two most common congeners, having been identified in 91% and 78% of lakes, respectively. MCLA, MCLY, and MCYR were also common, being identified in 52%, 61%, and 65% of the lakes, respectively. A key finding from this and other studies is that MCs rarely occur as single congeners in blooms. The levels of MCLA identified in the Midwestern lakes ranged from 0.05 to 54.0 µg/L and the average, exclusive of the highest level, was 0.39 µg/L. A level of MCLA 54.0 µg/L was found in a lake where the levels of MCLR and MCRR were 13,000 and 16,000 µg/L, respectively. Toxicity from MCs in this lake would therefore be due to a mixture of the most toxic congeners, MCLR and MCLA. These data presented in this paper indicate that the relative toxicities of the different congeners significantly differ, and that the observed congener-specific type of toxicity, exemplified by MCLA-induced icterus, indicates the probability that there are congener-specific differences in mechanisms of action. These findings indicate the need for future research efforts on potential HAB toxicities.

## 4. Materials and Methods

### 4.1. Animals

Equal numbers of male and female (10- to 12-week-old) BALB/c mice were obtained from Charles River Laboratories (Raleigh, NC, USA). After arrival of the animals at the Center for Public Health and Environmental Assessment animal facility, they were acclimated for at least 5 days prior to dosing. Animals were housed three per cage by sex in polycarbonate cages on heat-treated pine shaving bedding. Cages were placed on the racks in random positions. The animal rooms had a controlled temperature range (22–26 °C) and a 12:12 h light–dark cycle. Animals were fed commercial rodent chow (Purina Prolab) and filtered (5 µm) municipal tap water ad libitum. All studies were conducted after approval by the Institutional Animal Care and Use Committee of the CPHEA (Laboratory Animal Protocol for Research #18-09-002; approved 10-2-15), using recommendations of the 2011 NRC “Guide for the Care and Use of Laboratory Animals”, and the Public Health Service Policy on the Humane Care and Use of Laboratory Animals [57].

### 4.2. Compounds

The MCs used for dosing solutions were obtained from Enzo Life Sciences (MCLR, MCLY, MCYR) or Beagle Bioproducts (MCLA, MCRR). MCs were reconstituted into stock solutions using Picopure^®^ water at a target concentration of 1 g/L. To ensure 100% reconstitution of the dry MCs, stock solutions were sonicated for one hour, followed by overnight refrigeration and an additional hour of sonication. Individual dosing solutions were prepared for each dosing group by diluting stock solutions with Picopure^®^ water. Dosing concentrations were calculated assuming a 0.2 mL dose volume, or 0.4 mL in the case of high MCRR dose levels, and a mouse weight equivalent to the average weight of mice in each group being dosed.

The concentration of each stock solution was verified using an Agilent 6210 series time of flight mass spectrometer (MS-TOF) coupled to an Agilent 1100 series liquid chromatograph (LC). MC stock solutions were quantified against a reference material purchased from an independent vendor for each MC with two separate methods (i.e., external calibration curves and standard addition curves). All stock solutions were verified to be within 25% of the target concentrations except for MCRR (Table 1). For MCRR, the concentration in the stock solution was verified to be lower than the target, so it was assumed to be 0.85 g/L in dosing solution preparation.

### 4.3. Dosing, Animal Observation, and Necropsy

The range of dose levels administered were based on a previous acute toxicity study in our laboratory that evaluated toxic response in adult animals exposed to a single oral 7 mg/kg dose of these microcystin congeners [48]. The dosing scheme in this current study involved selecting initial dose levels based upon the severity of responses to the previous 7 mg/kg exposures, then adjusting dose levels as needed to determine NOAELs and LOAELs. Doses were administered by gavage a single time.

Animals were weighed the day prior to dosing and randomly assigned to treatment groups. All compounds were tested in three blocks of animals purchased from the vendor at least one week apart over a period of three months. MCs were administered by gavage using a 20 g feeding needle (Perfectum^®^, New Hyde Park, NY, USA). Immediately after dosing, animals were placed in metabolism cages to allow urine to be collected for MC quantification [58]. The number of animals (equal numbers of males and females) ranged from 12 to 29 per MC congener dose group except for the 22 mg/kg MCRR animals that were treated once and had a total of 6 animals. The MC-treated animals received the toxin in 0.2 mL of Picopure^®^ water and controls received 0.2 mL of Picopure^®^ water alone except for the 22 mg/kg MCRR group that received 0.4 mL. Animals which required a dose of 0.4 mL were given two 0.2 mL doses one hour apart. Animals were dosed in the morning and placed in metabolism cages. They were monitored for the initial 1/2 h post-dosing, at hourly intervals for the following six hours, and for three hours the following morning. All animals were euthanized at the end of this period, 24 h post-dosing. Animals displaying moribundity, defined as hunching, non-responsiveness to interaction, inappetence and/or lethargy, hypothermia, diarrhea, and/or weight loss greater than 10% were removed from the study, euthanized, necropsies performed, and blood and tissues were collected for analysis.

All necropsies were performed with observers blind to treatment. Animals were anesthetized by CO_2_ inhalation, weighed and euthanized by exsanguination by cardiac puncture with a 25 g, 5/8 in. needle attached to a 1 mL syringe. Whole blood for clinical chemistry was transferred to a 0.5 mL serum separator tube, allowed to clot for approximately 1 h, and centrifuged at 1300× *g* for 2 min to separate the serum. Immediately after blood collection, a gross assessment of liver appearance was recorded and a liver score was assessed based on presence/severity of lesion (possible score 0–2) and extent of liver surface area affected by the lesion (possible score 1–3). Lesions were the visual anomalies of congestion/hemorrhage, infiltration (of glycogen or lipid), or a reticulated pattern (pronounced pattern of the lobules). The scores given to individual animals were in the range 1–16, and normal was considered a score of 0–2; mild 3–5; moderate 6–8; and severe ≥9. A maximum score of 15 is possible based on gross lesions, but a score of 16 was automatically assigned if the animal was moribund due to hepatic hemorrhage and required euthanasia prior to the 24 h timepoint. The liver was removed, weighed, and placed in buffered formalin for future genomic and histological analyses. Spleen, pancreas, kidneys, duodenum, jejunum, ileum, testes/ovaries and uterus, lungs, heart, and thymus were also placed in 10% neutral buffered formalin for histopathology and these data will be presented in the future.

### 4.4. Clinical Chemistry

All serum clinical chemistry analyses were carried out using the Randox Daytona Plus instrument (Belfast, UK). Hepatocellular injury was assessed by determining the serum activities of ALT, AST, GLDH, and bilirubin. Markers of potential renal injury included serum concentrations of blood urea nitrogen (BUN) and creatinine. Serum glucose, total protein, and albumin were also measured as markers of general toxicity. All assays were performed using reagents obtained from the instrument manufacturer.

### 4.5. Statistical Analysis

All statistical analyses were conducted using SASv9.4 [59]. All variables were analyzed separately by sex. For each variable, an overall test to demonstrate that there were no differences in means across the treatment groups was performed. If this hypothesis was rejected (*p* ≤ 0.05), then each treatment group was compared with the control group and *p* ≤ 0.05 was considered statistically significant. For this reason, the pattern of significance across congeners and variables should be emphasized to a greater extent than individual *p*-values.

Continuous variables were analyzed with mixed-effects linear models (SAS Proc Mixed), essentially one-way analysis of variance (ANOVA), looking for any effect to determine the significance for each congener-associated effect. Block was included as a random effect in the model to address any block-to-block variability. For cases where two samples were combined to obtain enough volume for measurement, the observations were assigned a weight of 2 for clinical chemistry values; all others were assigned a weight of 1. Levene’s test (Proc GLM) and the Shapiro–Wilk test (Proc Univariate) were used to examine homogeneity of variance and normality of the data on both the log (base 10) and linear (original) scales. If the log scale improved these properties, the variable was analyzed on the log scale; otherwise the linear scale was used. For continuous variables, the F test of treatment group effect was used to determine whether there were differences among the groups, and if *p* ≤ 0.05, pairwise *t*-tests relative to control were performed for each congener.

For the categorical analyses of the liver score and percent dead/moribund, there were not sufficient sample sizes to include block in the analysis. Liver score was analyzed with the mixed-model ANOVA described above as well as a categorical analysis. The Cochran–Mantel–Haenszel (CMH) statistic testing for differences among group mean scores was used for the overall test of treatment for liver score, and the CMH trend test for individual comparisons of congener with control. For percent dead/moribund, a Pearson chi-square was used as the overall test and Fisher’s exact tests for the individual comparisons. The categorical analyses were conducted in Proc Freq.

## Figures and Tables

**Table 1:** Microcystin congener no observed adverse effect levels (NOAELs), lowest adverse effect levels (LOAELs), and types of LOAEL effects. ^a^

Congener	NOAEL (mg/kg)	LOAEL (mg/kg)	LOAEL Effects
MCLA	1	3	↑wt loss ^b^; ↑L/BW; ↑ALT; ↑AST; ↑GLDH; ↑liver score; ↑BUN/creatinine; ↓glucose; ↑total bilirubin
MCLR	3	5	↑wt loss; ↑L/BW; ↑ALT; ↑AST; ↑GLDH; ↑liver score; ↓glucose
MCLY	3	5	↑wt loss; ↑L/BW; ↑ALT; ↑GLDH; ↑liver score; ↓glucose
MCRR	9	22	↑wt loss; ↓glucose
MCYR	5	7	↑wt loss; ↑liver score; ↑BUN/creatinine; ↓glucose

^a^ Effects listed below were significantly different from controls, *p* ≤ 0.01. Liver score by Cochran–Mantel–Haenszel chi-square; all other endpoints by ANOVA/*t*-test. ^b^ Significant weight decreases during the time period between dosing and necropsy 24 h later.

## Supplementary Materials

The following are available online at https://www.mdpi.com/2072-6651/13/2/86/s1, Table S1. Summary of data for MCLA; Table S2. Summary of data for MCLR; Table S3. Summary of data for MCLY; Table S4. Summary of data for MCRR; Table S5. Summary of data for MCYR.
