# Supplementary material for: Dose–Response Study of Microcystin Congeners MCLA, MCLR, MCLY, MCRR, and MCYR Administered Orally to Mice
Source: Toxins (Basel). 2021 Jan 24;13(2):86. doi: 10.3390/toxins13020086 (PMC7911753; doi:10.3390/toxins13020086)
Supplement: Supplementary file 1 [file toxins-13-00086-s001.pdf]

Supplementary Materials: Dose-Response Study of Microcystin Congeners MCLA, MCLR, MCLY, MCRR, and MCYR Administered Orally to Mice

Neil Chernoff, Donna Hill, Johnsie Lang, Judith Schmid, Amy Farthing and Hwa Huang

Table S1. Summary of data for MCLA[1].

Male

Dose	Control	0.5 mg/kg	1 mg/kg	3 mg/kg	7 mg/kg
Number of animals	24	12	12	6	8
Moribundity (%)	0	0	0	0	100.0 ***
Weight Change (g.)	−1.89 ± 0.15	−2.26 ± 0.20	−1.64 ± 0.20	−2.63 ± 0.26 **	−1.42 ± 0.24
Liver weight (g.)	1.07 ± 0.02	1.13 ± 0.03	1.12 ± 0.03	1.17 ± 0.05	1.88 ± 0.04 ***
Liver/body weight	4.97 ± 0.10	5.02 ± 0.14	5.09 ± 0.14	5.78 ± 0.18 ***	8.96 ± 0.17 ***
ALT (log_{10}(IU/L))	1.76 ± 0.06	1.65 ± 0.08	1.67 ± 0.08	3.47 ± 0.12 ***	4.05± 0.17 ***
AST (log10(IU/L))	2.06 ± 0.06	1.98 ± 0.08	2.01 ± 0.08	3.51 ± 0.12 ***	3.84 ± 0.16 ***
GLDH (log10(IU/L))	1.28 ± 0.03	1.22 ± 0.05	1.17 ± 0.05	2.37 ± 0.08 ***	2.44 ± 0.09 ***
Liver score	2.92 ± 0.41	4.18 ± 0.62	3.00 ± 0.43	9.67 ± 0.33 **	16.00 ± 0.00 ***
BUN (mg/dL)	8.69 ± 0.66	10.19 ± 0.81 *	9.07 ± 0.81	8.26 ± 0.97	13.24 ± 1.27 ***
Cr (mg/dL)	0.50 ± 0.02	0.49 ± 0.03	0.50 ± 0.03	0.42 ± 0.03 **	0.53 ± 0.04
BUN_Cr	17.63 ± 1.03	20.22 ± 1.36 *	17.56 ± 1.36	19.08 ± 1.72	24.65 ± 2.31**
Albumin (g/dL)	3.65 ± 0.09	3.70 ± 0.11	3.63 ± 0.11	3.63 ± 0.14	2.83 ± 0.19 ***
Globulin(g/dL)	2.39 ± 0.39	2.44 ± 0.40	2.23 ± 0.40	2.16 ± 0.41	1.62 ± 0.43 ***
Total protein (g/L)	6.04 ± 0.40	6.17 ± 0.43	5.89 ± 0.43	5.81 ± 0.45	4.43 ± 0.52 ***
Glucose (mg/dL)	238.33 ± 6.56	185.60 ± 9.28 ***	228.04 ± 9.28	110.01 ± 13.13 ***	124.09 ± 18.57 ***
Total bilirubin (mg/dL)	0.12 ± 0.12	0.11 ± 0.16	0.07 ± 0.16	2.02 ± 0.24 ***	1.12 ± 0.31**

Female

Dose	Control	0.5 mg/kg	1 mg/kg	3 mg/kg	7 mg/kg
Number of animals	24	12	12	6	8
Moribundity (%)	0	0	0	0	62.5 **
Weight Change (g.)	−1.41 ± 0.08	−1.37 ± 0.11	−1.43 ± 0.11	−2.47 ± 0.16 ***	−2.06 + 0.14 ***
Liver weight (g.)	0.80 ± 0.03	0.83 ± 0.04	0.88 ± 0.04 *	0.97 ±0.05 ***	1.21 + 0.04 ***
Liver/body weight	4.60 ± 0.11	4.64 ± 0.15	4.83 ± 0.15	6.04 ± 0.19 ***	7.25 ± 0.16 ***
ALT (log_{10}(IU/L))	1.89 ± 0.13	2.05 ± 0.18	1.85 ± 0.18	3.37 ± 0.24 ***	3.90 ± 0.21 ***
AST (log10(IU/L))	2.26 ± 0.08	2.42 ± 0.12	2.21 ± 0.12	3.58 ± 0.16 ***	4.38 ± 0.17 ***
GLDH (log10(IU/L))	1.39 ± 0.09	1.48 ± 0.13	1.33 ± 0.13	1.82 ± 0.17 *	1.79 ± 0.18 *
Liver score	1.00 ± 0.36	2.50 ± 0.54	3.67 ± 0.59	9.50 ± 0.22 **	13.75 ± 1.40 ***
BUN (mg/dL)	8.91 ± 0.54	8.78 ± 0.77	8.77 ± 0.77	8.89 ± 1.07	11.88 ± 0.95 **
Cr (mg/dL)	0.51 ± 0.02	0.50 ± 0.03	0.50 ± 0.03	0.41 ± 0.04 **	0.39 ± 0.03 ***
BUN_Cr	17.54 ± 0.77	18.17 ± 1.09	17.99 ± 1.09	21.63± 1.55 *	29.24 ± 1.43 ***
Albumin (g/dL)	3.76 ± 0.25	3.66 ± 0.32	3.78 ± 0.32	3.43 ± 0.39	4.46 ± 0.34 *
Globulin(g/dL)	2.20 ± 0.42	2.28 ± 0.43	2.28 ± 0.43	2.14 ±0.44	1.97 ± 0.44
Total protein (g/L)	5.91 ± 0.41	5.98 ± 0.48	6.09 ± 0.48	5.67 ± 0.56	6.42 ± 0.50
Glucose (mg/dL)	208.76 ± 12.86	199.74 ± 16.15	213.46 ± 16.15	102.60 ± 19.65 ***	88.50 ± 17.45 ***
Total bilirubin (mg/dL)	0.12 ± 0.15	0.10 ± 0.19	0.15 ± 0.20	2.72 ± 0.30 ***	4.33 ± 0.25 ***

All significance tests of difference from control. Mortality statistical significance by Fisher's exact test; liver score by Cochran-Mantel-Haenszel chi-square; * $p \le 0.05$ for differences from controls; ** $p \le 0.01$ for differences from controls; *** $p \le 0.001$ for differences from controls.

Table S2. Summary of data for MCLR[1.]

MCLR

Male

Dose	Control	0.5 mg/kg	1 mg/kg	3 mg/kg	5 mg/kg	7 mg/kg	9 mg/kg	11 mg/kg
Number of animals	24	6	6	12	6	14	6	6
Moribundity (%)	0	0	0	0	33.3	28.6 *	100.0 **	100.0 **
Weight Change (g.)	−1.96 ± 0.24	−2.01 ± 0.38	−1.90 ± 0.38	−2.01± 0.29	−2.96 ± 0.35 **	−2.74±0.27 **	−1.67 ± 0.37	−1.08 ± 0.35 **
Liver weight (g.)	1.03 ± 0.04	1.09 ± 0.09	1.23 ± 0.09 *	1.07 ± 0.06	1.29 ± 0.08 **	1.25±0.06 **	1.69 ± 0.09 ***	1.82 ± 0.08 ***
Liver/body weight	5.02 ± 0.25	5.16 ± 0.45	5.31 ± 0.43	5.24 ± 0.31	6.58 ± 0.39 ***	6.54±0.29 ***	8.33± 0.42 ***	8.81 ± 0.39 ***
ALT (log_{10}(IU/L))	1.76 ± 0.16	1.82 ± 0.28	1.80 ± 0.28	2.09 ± 0.21	2.98 ± 0.26***	3.31±0.19 ***	3.68 ± 0.30 ***	3.31 ± 0.28 ***
AST (log10(IU/L))	2.08 ± 0.12	2.14 ± 0.22	2.01 ± 0.22	2.24 ± 0.16	2.76 ± 0.20**	2.91±0.14 ***	3.22 ± 0.24 ***	2.91 ±0.22 ***
GLDH (log10(IU/L))	1.34 ± 0.11	1.41 ± 0.19	1.32 ± 0.19	1.52 ± 0.14	1.86 ± 0.18**	2.14±0.13 ***	2.22 ± 0.20 * **	1.70 ± 0.19
Liver score	3.79± 0.68	5.50 ± 0.92	5.33 ± 0.76	4.42 ± 1.16	11.00 ± 2.53**	12.07±0.97 ***	15.83 ± 0.17 **	16.00 ± 0.00 **
BUN (mg/dL)	9.28 ± 0.33	9.87 ± 0.66	9.97 ± 0.66	10.06 ± 0.46	9.53 ± 0.66	10.58 ± 0.45 *	9.25 ± 0.80	10.53 ± 0.72
Cr (mg/dL)	0.46 ± 0.03	0.50 ± 0.04	0.50±0.04	0.48±0.03	0.44 ± 0.04	0.45 ± 0.03	0.39 ± 0.04	0.52 ± 0.04
BUN_Cr	20.63 ± 1.72	19.52 ± 3.23	19.81±3.23	21.44±2.30	24.20±3.02	24.00 ± 2.11	35.86 ± 3.58 ***	21.04 ± 3.25
Albumin (g/dL)	3.45 ± 0.16	3.68 ± 0.23	3.71 ± 0.23	3.64 ± 0.19	3.44 ± 0.22	3.41 ± 0.17	2.51 ± 0.25 ***	3.05 ± 0.23
Globulin(g/dL)	1.93 ± 0.18	2.17 ± 0.22	2.22 ± 0.22	2.12 ± 0.19	1.88 ± 0.21	1.88 ± 0.18	1.43 ± 0.23 **	1.78 ± 0.22
Total protein (g/L)	5.42 ± 0.24	5.85 ± 0.36	5.93 ± 0.36	5.72 ± 0.28	5.24 ±0.34	5.30 ± 0.26	3.88 ± 0.39 ***	4.74 ± 0.36 *
Glucose (mg/dL)	192.76 ± 15.57	231.82 ± 26.02	188.78 ± 26.02	211.98 ± 19.52	153.58 ± 24.25	102.58 ± 18.92 ***	157.90 ± 28.03	168.82 ± 25.79
Total bilirubin (mg/dL)	0.16 ± 0.04	0.12 ± 0.06	0.18 ± 0.06	0.15 ± 0.05	0.35 ± 0.07 **	0.29 ± 0.04 **	0.21 ± 0.07	0.33 ±0.06 **

Female

Dose	Control	0.5 mg/kg	1 mg/kg	3 mg/kg	5 mg/kg	7 mg/kg	9 mg/kg	11 mg/kg
Number of animals	24	6	6	12	6	15	9	6
Moribundity (%)	0	0	0	0	0	6.7	100.0 **	100.0 **
Weight Change (g.)	−1.60 ± 0.11	−1.69 ± 0.20	−1.31 ± 0.20	−1.50 ± 0.14	−2.59 ± 0.19	−2.69 ± 0.13 ***	−0.45± 0.17 ***	−0.89 ± 0.21 **
Liver weight (g.)	0.81 ± 0.03	0.89 ± 0.05	0.87 ± 0.05	0.82 ± 0.04	0.81 ± 0.05	0.92 ± 0.03 **	1.44 ± 0.04 ***	1.39 ± 0.05 ***
Liver/body weight	4.60 ± 0.18	4.87 ± 0.29	4.65 ± 0.29	4.75 ± 0.22	4.98 ± 0.27	5.62 ± 0.20 ***	8.04 ± 0.24 ***	7.61 ± 0.27 ***
ALT (log_{10}(IU/L))	1.87 ± 0.13	1.69 ± 0.31	1.85 ± 0.26	1.90 ± 0.18	2.94 ± 0.25 ***	3.41 ±0.16 ***	3.35 ± 0.25 ***	3.38 ±0.29 ***
AST (log10(IU/L))	2.24 ± 0.11	1.95 ± 0.26	2.14 ± 0.22	2.20 ± 0.15	2.80 ± 0.21 *	3.17 ± 0.13 ***	3.22 ± 0.21 ***	3.23 ± 0.26 ***
GLDH (log10(IU/L))	1.40 ± 0.10	1.08 ± 0.22	1.31 ± 0.19	1.37 ± 0.13	2.05 ± 0.18 **	2.31 ± 0.12 ***	1.78 ± 0.18	1.61 ± 0.21
Liver score	0.79 ± 0.32	3.83 ± 0.17	3.83 ± 0.17	1.83 ± 0.51	9.00 ± 1.21 **	9.20 ± 0.82 ***	16.00 ± 0.00 ***	16.00 ± 0.00 **
BUN (mg/dL)	8.55 ± 0.61	8.04 ± 1.36	9.41 ± 1.17	9.08 ± 0.83	9.41 ± 1.10	10.66 ± 0.72 *	9.88 ± 1.11	11.41 ± 1.30 *

Cr (mg/dL)	0.49 ± 0.03	0.43 ± 0.05	0.49 ± 0.04	0.48 ± 0.03	0.49 ± 0.04	0.49 ± 0.03	0.51 ± 0.04	0.52 ± 0.04
BUN_Cr	17.25 ± 0.99	18.76 ± 2.24	18.80 ± 1.92	18.11 ± 1.34	20.41 ± 1.80	22.24 ± 1.18 ***	21.89 ± 1.81 *	24.06 ± 2.14 **
Albumin (g/dL)	3.73 ± 0.16	3.29 ± 0.29	3.51 ± 0.26	3.86 ± 0.20	3.89 ± 0.24	3.69 ± 0.18	2.93 ± 0.25 **	3.12 ± 0.28 *
Globulin(g/dL)	1.83 ± 0.22	1.50 ± 0.27	1.66 ± 0.26	1.89 ± 0.23	1.92 ± 0.25	1.67 ± 0.22	1.43 ± 0.25 *	1.45 ± 0.26 *
Total protein (g/L)	5.52 ± 0.27	4.78 ± 0.47	5.15 ± 0.42	5.73 ± 0.33	5.79 ± 0.39	5.36 ± 0.30	4.34 ± 0.39 **	4.55 ± 0.44 *
Glucose (mg/dL)	211.88 ± 16.16	176.12 ± 38.41	222.87 ± 32.37	164.91 ± 22.19	104.46 ± 30.98 **	100.47 ± 19.79 ***	217.97 ± 31.12	135.09 ± 37.25
Total bilirubin (mg/dL)	0.17 ± 0.05	0.19 ± 0.09	0.17 ± 0.08	0.18 ± 0.06	0.17 ± 0.07	0.20 ± 0.05	0.32 ± 0.07 *	0.20 ± 0.08

All significance tests of difference from control. Mortality statistical significance by Fisher's exact test; liver score by Cochran-Mantel-Haenszel chi-square; * $p \le 0.05$ for differences from controls; ** $p \le 0.01$ for differences from controls; *** $p \le 0.001$ for differences from controls.

Table S3. Summary of data for MCLY[1].

Male

Dose	Control	1 mg/kg	3 mg/kg	5 mg/kg	7 mg/kg
Number of animals	27	6	14	13	8
Moribundity (%)	0	0	0	0	25
Weight Change (g.)	−2.01 ± 0.16	−1.31 ± 0.31 *	−1.74 ± 0.21	−2.72 ± 0.21 **	−2.18 ± 0.26
Liver weight (g.)	1.09 ± 0.08	0.89 ± 0.12	1.09 ± 0.09	1.15 ± 0.09	1.45 ± 0.10 ***
Liver/body weight	5.01 ± 0.28	4.66 ± 0.43	4.94 ± 0.33	5.39 ± 0.24	6.67 ± 0.37 ***
ALT (log_{10}(IU/L))	1.64 ± 0.07	1.83 ± 0.15	1.63 ± 0.10	2.08 ± 0.10 ***	2.38± 0.15 ***
AST (log10(IU/L))	2.02 ± 0.05	2.04 ± 0.11	1.90 ± 0.07	2.25 ± 0.07 *	2.44 ± 0.11 **
GLDH (log10(IU/L))	1.22 ± 0.06	1.32 ± 0.13	1.21 ± 0.09	1.65 ± 0.09 ***	1.95 ± 0.13 ***
Liver score	3.48 ± 0.51	3.00 ± 1.00	1.93 ± 0.54 *	5.62 ± 0.98	7.75 ± 2.28 *
BUN (mg/dL)	8.92 ± 0.40	9.27 ± 0.85	9.93 ± 0.56	9.31 ± 0.58	10.51 ± 0.82
Cr (mg/dL)	0.52 ± 0.05	0.49 ± 0.06	0.51 ± 0.05	0.51 ± 0.05	0.50 ± 0.06
BUN_Cr	17.64 ± 1.37	21.31 ± 2.00	19.70 ± 1.61	19.89 ± 1.60	20.30 ± 1.88
Albumin (g/dL)	3.57 ± 0.08	3.58 ± 0.15	3.62 ± 0.11	3.62 ± 0.11	3.57± 0.14
Globulin(g/dL)	2.05 ± 0.17	1.95 ± 0.19	2.11 ± 0.18	2.11 ± 0.18	1.95 ± 0.19
Total protein (g/L)	5.58 ± 0.13	5.54 ± 0.25	5.70 ± 0.17	5.72 ± 0.18	5.53 ± 0.23
Glucose (mg/dL)	205.32 ± 7.60	212.96 ± 16.09	202.53 ± 10.51	151.87 ± 10.86 ***	130.77 ± 15.49 ***
Total bilirubin (mg/dL)	0.23 ± 0.07	0.01 ± 0.10 **	0.21 ± 0.08	0.26 ± 0.08	0.22 ± 0.09

Female

Dose	Control	1 mg/kg	3 mg/kg	5 mg/kg	7 mg/kg
Number of animals	27	6	14	14	8
Moribundity (%)	0	0	0	0	0
Weight Change (g.)	−1.41 ± 0.10	−1.20 ± 0.21	−1.57 ± 0.14	−1.90 ± 0.14	−2.53 ±0.18 ***
Liver weight (g.)	0.83 ± 0.04	0.78 ± 0.05	0.88 ± 0.04	0.95± 0.04 ***	0.94 ± 0.05 **
Liver/body weight	4.64± 0.16	4.35 ± 0.26	4.86 ±0.19	5.18 ± 0.19 **	5.72 ± 0.22 ***
ALT (log_{10}(IU/L))	1.87 ± 0.13	1.46 ± 0.26	1.98 ± 0.18	2.07 ± 0.18	2.41 ± 0.22 *
AST (log10(IU/L))	2.26 ± 0.09	1.98 ± 0.18	2.31 ± 0.12	2.38 ± 0.12	2.75 ± 0.16 **
GLDH (log10(IU/L))	1.33 ± 0.10	1.00 ± 0.19	1.42 ± 0.13	1.65 ± 0.13 *	1.70 ± 0.16 *
Liver score	0.85 ± 0.30	1.67 ± 1.09	2.36 ± 0.62	5.14 ± 0.74 **	5.50 ± 1.59 **
BUN (mg/dL)	9.21 ± 0.49	9.46 ± 0.91	7.71 ± 0.64 *	8.61 ± 0.64	8.68 ± 0.76
Cr (mg/dL)	0.55 ± 0.06	0.56 ± 0.07	0.54± 0.06	0.55 ± 0.06	0.48 ± 0.07
BUN_Cr	17.09 ± 1.30	17.93 ± 2.02	14.71 ± 1.57	32.76 ± 18.71	33.08 ± 20.56
Albumin (g/dL)	3.78 ± 0.12	3.76 ± 0.20	3.82 ± 0.15	3.72 ± 0.15	3.46 ± 0.17 *
Globulin(g/dL)	1.86 ± 0.22	1.89 ± 0.25	1.85 ± 0.23	1.89 ± 0.23	1.58 ± 0.24 *
Total protein (g/L)	5.59 ± 0.23	5.64 ± 0.34	5.66 ± 0.27	5.59 ± 0.27	5.07 ± 0.30 *
Glucose (mg/dL)	198.85 ± 13.27	234.2 ± 23.53	220.22 ± 16.90	177.18 ± 16.90	133.22 ± 19.74 **
Total bilirubin (mg/dL)	0.18 ± 0.16	0.02 ± 0.28	0.26 ± 0.21	0.59 ± 0.20 *	0.59 ± 0.23

All significance tests of difference from control. Mortality statistical significance by Fisher's exact test; liver score by Cochran-Mantel-Haenszel chi-square; * $p \leq 0.05$ for differences from controls; ** $p \leq 0.01$ for differences from controls; *** $p \leq 0.001$ for differences from controls.

Table S4. Summary of data for MCRR[1.]

Male

Dose	Control	7 mg/kg	9 mg/kg	11 mg/kg	22 mg/kg
Number of animals	21	9	6	9	3
Moribundity (%)	0	0	0	0	0
Weight Change (g.)	−1.97 ± 0.17	−1.92 ± 0.22	−1.50 ± 0.29	−2.26 ± 0.24	−2.79 ± 0.35 *
Liver weight (g.)	1.02 ± 0.01	1.03 ± 0.02	1.11± 0.03 **	1.09 ± 0.02 *	0.92 ± 0.04 *
Liver/body weight	4.88 ± 0.10	5.01 ± 0.12	5.22 ± 0.15 *	5.02 ± 0.13	4.77 ± 0.18
ALT (log_{10}(IU/L))	1.65 ± 0.06	1.89 ± 0.08 *	1.54 ± 0.10	1.55 ± 0.08	1.65 ± 0.15
AST (log10(IU/L))	2.04 ± 0.04	2.17 ± 0.07	1.88 ± 0.08	1.96 ± 0.07	2.08 ± 0.11
GLDH (log10(IU/L))	1.22 ± 0.04	1.34 ± 0.05 *	1.13 ± 0.07	1.23 ± 0.05	1.30 ± 0.09
Liver score	3.00 ± 0.40	3.00 ± 0.58	3.83 ± 0.31	3.89 ± 0.48	4.00 ± 0.00
BUN (mg/dL)	9.46 ± 0.28	9.45 ± 0.43	7.21 ± 0.53 ***	8.24 ± 0.43 *	9.12 ±0.74
Cr (mg/dL)	0.55 ± 0.05	0.55 ± 0.05	0.47 ± 0.06 *	0.48 ± 0.05 *	0.53 ± 0.06
BUN_Cr	17.7 ± 1.29	17.29 ± 1.44	15.50 ± 1.68	17.39 ± 1.52	17.54 ± 1.85
Albumin (g/dL)	3.61 ± 0.08	3.54 ± 0.11	3.44 ± 0.14	3.58 ± 0.12	3.90 ± 0.18
Globulin(g/dL)	2.03 ± 0.21	1.92 ± 0.21	2.00 ± 0.23	2.22 ± 0.22	2.17 ± 0.24
Total protein (g/L)	5.62 ± 0.19	5.44 ± 0.24	5.49 ± 0.30	5.83 ± 0.25	6.10 ± 0.34
Glucose (mg/dL)	216.88 ± 8.75	224.05 ± 12.60	218.70 ± 16.36	215.71± 13.24	183.48 ± 21.35
Total bilirubin (mg/dL)	0.18 ± 0.06	0.15± 0.08	0.28 ± 0.09	0.26 ± 0.08	0.25 ± 0.12

Female

Dose	Control	7 mg/kg	9 mg/kg	11 mg/kg	22 mg/kg
Number of animals	21	8	6	9	3
Moribundity (%)	0	0	0	0	0
Weight Change (g.)	−1.30 ± 0.09	−1.51 ± 0.13	−1.89 ± 0.17 **	−1.67 ± 0.14 *	−2.16 ± 0.21 ***
Liver weight (g.)	0.85± 0.04	0.85 ± 0.04	0.75 ± 0.05 **	0.82 ± 0.04	0.89 ± 0.05
Liver/body weight	4.73 ± 0.18	4.84 ± 0.19	4.21 ± 0.21 ***	4.51 ± 0.20 *	4.95 ± 0.22
ALT (log_{10}(IU/L))	1.81 ± 0.07	1.79 ± 0.12	1.60 ± 0.14	1.76 ± 0.11	1.76 ± 0.19
AST (log10(IU/L))	2.24 ± 0.06	2.16 ± 0.10	2.17 ± 0.12	2.08 ± 0.10	1.98 ± 0.17
GLDH (log10(IU/L))	1.29 ± 0.05	1.34 ±0.09	1.19 ± 0.10	1.26 ± 0.08	1.27 ± 0.14
Liver score	0.95 ± 0.38	1.25±0.84 **	3.33 ± 1.05	2.11 ± 0.68	5.33 ± 1.33
BUN (mg/dL)	9.34 ± 0.65	8.61 ± 0.85	6.90 ± 1.10 *	7.42± 0.88 *	6.10 ± 1.24 **
Cr (mg/dL)	0.58 ± 0.07	0.53 ±0.07	0.57 ± 0.08	0.61 ±0.07	0.62 ± 0.08
BUN_Cr	16.19 ± 1.20	16.89 ±1.46	22.90 ± 23.74	21.73 ± 21.53	15.62 ± 25.83
Albumin (g/dL)	3.87 ± 0.09	3.66 ± 0.14	3.57 ± 0.17	3.70 ±0.14	3.94 ± 0.23
Globulin(g/dL)	1.88 ± 0.27	1.61 ± 0.28 *	1.81 ± 0.30	1.89 ±0.28	2.05 ± 0.31
Total protein (g/L)	5.69 ± 0.25	5.25 ± 0.30	5.49 ±0.36	5.67 ± 0.32	6.02 ± 0.41
Glucose (mg/dL)	214.08 ± 11.46	205.47 ± 15.85	203.03 ± 19.86	224.90 ± 16.37	164.52 ± 24.19 *
Total bilirubin (mg/dL)	0.16 ± 0.04	0.17 ± 0.07	0.29 ± 0.10	0.42 ± 0.06 **	0.30 ± 0.10

All significance tests of difference from control. Mortality statistical significance by Fisher's exact test; liver score by Cochran-Mantel-Haenszel chi-square; * $p \leq 0.05$ for differences from controls; ** $p \leq 0.01$ for differences from controls; *** $p \leq 0.001$ for differences from controls.

Table S5. Summary of data for MCYR[1.]

Male

Dose	Control	3 mg/kg	5 mg/kg	7 mg/kg	11 mg/kg
Number of animals	18	6	6	9	6
Moribundity (%)	0	0	0	11.1	33.3
Weight Change (g.)	−1.70 ± 0.20	−1.83 ± 0.34	−1.72 ± 0.34	−2.96 ± 0.28 ***	−2.67 ±0.34 *
Liver weight (g.)	1.11 ± 0.05	1.06 ± 0.09	1.11 ± 0.09	1.15 ± 0.07	1.49 ± 0.09 ***
Liver/body weight	5.05 ± 0.20	4.84 ± 0.33	5.07 ± 0.33	5.40 ± 0.26	6.66 ± 0.35 ***
ALT (log_{10}(IU/L))	2.02 ± 0.18	1.90 ± 0.25	2.07 ± 0.25	2.33 ± 0.21	3.36 ± 0.27 ***
AST (log10(IU/L))	2.21 ± 0.10	2.05 ± 0.15	2.27 ± 0.15	2.27 ± 0.12	2.94 ± 0.17 ***
GLDH (log10(IU/L))	1.39 ± 0.09	1.24 ± 0.15	1.50 ± 0.15	1.74 ± 0.12 **	2.35 ± 0.16 ***
Liver score	2.17 ± 0.49	2.00 ± 0.93	1.67 ± 1.05	7.89 ± 1.32 **	13.67 ± 0.92 **
BUN (mg/dL)	8.32 ± 1.23	6.25 ± 1.48 *	6.42 ± 1.48	11.47 ± 1.32 ***	11.49 ± 1.55 **
Cr (mg/dL)	0.49 ± 0.02	0.47 ± 0.03	0.49 ± 0.03	0.54 ± 0.03	0.52 ± 0.03
BUN_Cr	16.99 ± 2.02	14.10 ± 2.31 *	13.71 ± 2.31 *	20.95 ± 2.32 ***	21.96 ± 2.40 **
Albumin (g/dL)	3.61 ± 0.11	3.67 ± 0.16	3.69 ± 0.16	4.08 ± 0.13 ***	4.02 ± 0.18 *
Globulin(g/dL)	1.82 ± 0.20	1.86 ± 0.22	1.88 ± 0.22	2.02 ± 0.21 *	2.08 ± 0.22 *
Total protein (g/L)	5.41± 0.14	5.62 ± 0.22	5.66 ± 0.22	6.06 ± 0.18 **	6.18 ± 0.25 **
Glucose (mg/dL)	236.14 ± 7.45	196.00 ± 12.90 *	196.33 ± 12.90 *	145.21 ± 11.17 ***	85.47 ± 15.80 ***
Total bilirubin (mg/dL)	0.27 ± 0.16	0.27 ± 0.17	0.28 ± 0.17	0.38 ± 0.16*	0.41 ± 0.16 *

Female

Dose	Control	3 mg/kg	5 mg/kg	7 mg/kg	11 mg/kg
Number of animals	18	6	6	9	6
Moribundity (%)	0	0	0	0	16.7
Weight Change (g.)	−1.19 ± 0.14	−1.30 ± 0.25	−1.55 ± 0.30	−1.70 ± 0.20 *	−2.08 ± 0.25 **
Liver weight (g.)	0.88 ± 0.02	0.87 ± 0.04	0.98 ± 0.05	0.89 ± 0.03	1.02 ± 0.04 **
Liver/body weight	5.01 ± 0.09	4.75± 0.15	5.18 ± 0.19	5.35 ± 0.12 *	6.40 ± 0.15 ***
ALT (log_{10}(IU/L))	2.07 ± 0.22	1.87 ± 0.34	2.64 ± 0.38	2.33 ± 0.26	3.50 ± 0.32 ***
AST (log10(IU/L))	2.35 ± 0.15	2.09 ± 0.25	2.61 ± 0.29	2.59 ± 0.19	3.41 ± 0.24 ***
GLDH (log10(IU/L))	1.42 ± 0.12	1.36 ± 0.20	1.77 ±0.23	1.59 ± 0.15	2.30 ± 0.19 ***
Liver score	0.00 ± 0.00	0.00 ± 0.00	3.75 ± 2.39	3.89 ± 0.99 **	11.83 ± 1.17 **
BUN (mg/dL)	8.06 ± 0.85	9.05 ± 1.43	9.00 ± 1.75	9.39 ± 1.17	15.08 ± 1.32 ***
Cr (mg/dL)	0.53 ± 0.03	0.52± 0.04	0.50 ± 0.05	0.51 ± 0.03	0.49 ± 0.04
BUN_Cr	15.02 ± 1.99	15.86 ± 3.35	16.80 ± 4.11	18.51 ± 2.74	33.28 ± 3.11 ***
Albumin (g/dL)	3.96 ±0.11	3.84 ± 0.19	3.74 ± 0.23	3.84 ± 0.15	3.39 ± 0.18 **
Globulin(g/dL)	1.61 ± 0.25	1.66 ± 0.27	1.58 ± 0.27	1.57 ± 0.25	1.24 ± 0.26 **
Total protein (g/L)	5.53 ± 0.27	5.54 ± 0.39	5.35 ± 0.42	5.40 ± 0.31	4.61 ± 0.37 *
Glucose (mg/dL)	229.09 ± 19.81	199.66 ±25.72	150.26 ± 27.57 **	173.09± 21.50 **	76.13 ± 24.78 ***
Total bilirubin (mg/dL)	0.29 ± 0.12	0.27 ± 0.14	0.30 ± 0.15	0.27 ± 0.13	0.34 ± 0.14

All significance tests of difference from control. Mortality statistical significance by Fisher's exact test; liver score by Cochran-Mantel-Haenszel chi-square; * $p \leq 0.05$ for differences from controls; ** $p \leq 0.01$ for differences from controls; *** $p \leq 0.001$ for differences from controls.
